# Good to know – This is PPIE! Development of a training tool for public and patient involvement and engagement in pediatric oncological research

**DOI:** 10.1002/cnr2.1835

**Published:** 2023-05-11

**Authors:** Liesa Josephine Weiler‐Wichtl, Ulrike Leiss, Johannes Gojo, Anita Kienesberger, Rita Hansl, Maximilian Hopfgartner, Carina Schneider

**Affiliations:** ^1^ Department of Pediatrics and Adolescent Medicine, Comprehensive Center for Pediatrics and Comprehensive Cancer Center Medical University of Vienna Vienna Austria; ^2^ Childhood Cancer International – Europe (CCI‐E) Vienna Austria

**Keywords:** integrated care system, patient advocacy, patient and public involvement and engagement, patient‐oriented care, pediatric oncology, survivors

## Abstract

**Background:**

Public and Patient Involvement and Engagement (PPIE) in research is still a poorly understood and infrequently practiced concept, although the literature stresses clear benefits for quality of care and research as well as patient satisfaction and empowerment.

**Aim:**

The presently described project aimed at using different PPIE methods to evaluate the current state of knowledge about and attitude toward PPIE in research among different stakeholders of pediatric oncology in Europe. Based on the findings a tailored training tool directed toward the different stakeholders will be designed.

**Methods and Results:**

An interdisciplinary steering group developed a mixed‐method 3‐stage process to (1) investigate the current knowledge and attitudes about PPIE using a Europe‐wide cross‐sectional online survey directed toward health care professionals (*n* = 134) and the patient group (patients, survivors, family members, …) (*n* = 168). The results were analyzed quantitatively, focusing on group comparisons (t‐tests, *X* ^2^ tests). (2) In a live workshop with *n* = 36 participants (HCPs and patient group) dual moderation teams (HCPs and patient experts) guided the exploration of effective ways for practicing PPIE. Despite classifying PPIE as relevant, both HCPs and patients indicated a low level of knowledge about the concept and terminology (patients: *t*(334) = −2.82, *p* = .004; HCPs: *t*(270) = −2.88, *p* = .004). While HCPs assumed to already be involving patients in many research areas, this was not perceived by the patient group (*X* ^2^ (1, *N* = 304) = 42.70, *p* < .001). HCPs and patients named similar obstacles for implementing PPIE in research, though numerous creative solutions were found during the workshop (engagement). (3) The outcomes were integrated into a training tool (White‐Board movie).

**Conclusion:**

Although HCPs and patients acknowledge the benefit of PPIE, the presented results highlight the lack of awareness about the concept, and the need for effective tools for researchers to integrate PPIE throughout the entire research process, thereby contributing to a sustainable change within the scientific culture.

## 1 INTRODUCTION

Closing the gap between patients, researchers, and health care professionals (HCP) has been linked to better quality of care; higher real‐life relevance and translatability of studies; more successful recruitment for clinical trials; and more informed decisions as well as empowerment of those affected.^1^, ^2^, ^3^, ^4^, ^5^, ^6^, ^7^, ^8^, ^9^ To date, however, research priorities, decisions about funding allocation, and the conduct of scientific studies is predominantly guided by scientific researchers rather than the affected patient community.^10^, ^11^ Furthermore, information on current research projects as well as the results of clinical trials are often only accessible within the academic context and not presented in a language appropriate for lays.^12^ To counter this disparity, the UK National Institute for Health Research (NHIR) developed the concept of “Patient and Public Involvement and Engagement in Research” (PPIE). Defined as research “carried out ‘with’ or ‘by’ members of the public rather than ‘to’, ‘about’ or ‘for’ them,”^13^ PPIE aims at emancipating patients to become equivalent and competent members of and contributors to the health care system. By considering and valuing the perspective of those affected, the one unifying goal should be accomplished, namely optimizing care and research by ensuring that the patients' needs are met and their opinions are appreciated.^13^

Due to the multi‐facetted community and the great variety of stakeholders and research topics, PPIE can take various forms.^14^ On the one hand, there are different roles that members of the patient group can embrace, including “individual patients/carers” (person/family living with a disease), “patient advocates” (representing big(ger) groups of patients), “patient organization representatives,” and “patient experts” (individual patients with additional specific expertise).^15^ On the other hand, there are different ways and degrees to which the patient group can contribute. While participation (taking part in studies or clinical trials as study subjects) is an indispensable part of research, engagement (the dissemination of information and knowledge about research) and involvement (“active partnership between”^13^, ^16^ researchers and patients, patient representatives or the public throughout the research process) are less common.^17^, ^18^, ^19^ The choice of appropriate PPIE is especially relevant in pediatric oncology which is a field coined by the necessity of close interdisciplinary collaboration of various HCPs (doctors, nurses, psychologists, social workers, therapists, pedagogues, etc.) as well as a great variety of stakeholders on the patient side. These include patients, survivors, parents, other family members, patient organizations, and patient advocates, among others. Despite the increasing estimation of the benefits and value of engagement and involvement especially in the early stages of research, there is still insufficient implementation with clear regional differences.^5^, ^6^, ^9^, ^13^, ^20^ Globally there is a call for establishing PPIE as an integral part of research, although the appropriate and most beneficial form must be chosen on a project basis.^12^, ^14^

In addition to international differences and the lack of legal enforcement there are other obstacles on a national and institutional level that prevent the successful establishment of well‐practiced PPIE. These include language and socioeconomic barriers leading to miscommunication, poor recruitment strategies for patient representatives, a lack of financial and temporal resources, and deficient understanding and estimation of other stakeholders' perspectives.^5^, ^7^, ^17^, ^21^ Furthermore, there are different logics and rationales for practicing PPIE, leading to a risk for tokenism and the abuse of poorly practiced PPIE to legitimize managerial decisions rather than sincerely including patients' perspectives.^5^, ^16^, ^22^, ^23^, ^24^ To ensure that PPIE is not only established but also practiced well, organizations such as the UK Public Involvement Standards Development Partnership^25^ and the German Federal Ministry of Education and Research^26^ have published standards and principles of successful PPIE. Such guidelines are intended to serve as a reference point for researchers and governmental units as well as to foster reflection and improvement after failed attempts. The most dominant standards include the consideration of and accessibility for all relevant stakeholders, good communication between the subgroups, appreciative cooperation of all parties, and the efficient use of knowledge and resources.^13^, ^25^, ^26^, ^27^, ^28^

Of these standards especially the British National Institute for Health and Care Research (NIHR) Standards for Public Involvement^25^ and the guidelines by the former NIHR institute INVOLVE^28^ have been serving various researchers to evaluate their own efforts of practicing PPIE,^24^, ^29^, ^30^ as well as to create concrete frameworks, models, and quality guidance for successful PPIE.^3^, ^15^, ^29^, ^31^, ^32^, ^33^, ^34^, ^35^, ^36^ Various of these studies on PPIE do not only provide theoretical frameworks but also serve as best practice examples themselves since PPIE is both the subject of interest and part of the methodology, by involving patients and the public throughout the entire research process.^19^, ^29^, ^30^, ^33^, ^37^, ^38^, ^39^ In this context, review papers of studies implementing PPIE highlight the manifold opportunities for PPIE,^15^ with authors such as Bergerum et al. emphasizing the complexity of specific context variables that influence PPIE success,^40^ and Greenhalgh et al. hence pleading for each study to draw from evidence‐based resources to build their own framework for involvement.^36^ When reflecting upon the advantages and challenges of practicing PPIE in their studies, researchers like Aries et al. and Greenwood et al. conclude with the recommendation to plan PPIE as a fixed part at the beginning of the research project to achieve early involvement and full integration of at least one patient or member of the public in each step of the entire project, as well as sensible recruitment and task selection, appropriate remuneration of PPI advisors, clear and respectful communication and reasonable PPIE training for all stakeholders.^29^, ^30^, ^37^, ^41^, ^42^, ^43^, ^44^, ^45^

While the effectiveness of and methods for successful PPIE have accordingly become an increasingly popular research topic, the developed methods are predominantly directed toward adult patients, whereas methods for integrating children into the research process are still lacking.^46^ Furthermore, the awareness for and attitude toward PPIE among the various stakeholders in health care has only been investigated by few authors^12^ and an even lower number of studies has focused on the complex interdisciplinary field on pediatric oncology.^6^, ^12^, ^46^ The limited establishment of PPIE suggests that despite the considerable benefits for patient well‐being, health care quality and research relevance, the concept of PPIE is still uncommon, even in academic contexts.

Therefore, the research aims of the present study were to:

1. evaluate the current understanding of and the different attitudes toward the concept of PPIE among the various stakeholders of pediatric oncology in European countries, to then
2. design and pilot new PPIE practices as well as to
3. integrate the findings into a PPIE training tool realized as a short film.

## 2 METHODS

### 2.1 Design

**FIGURE 1:**
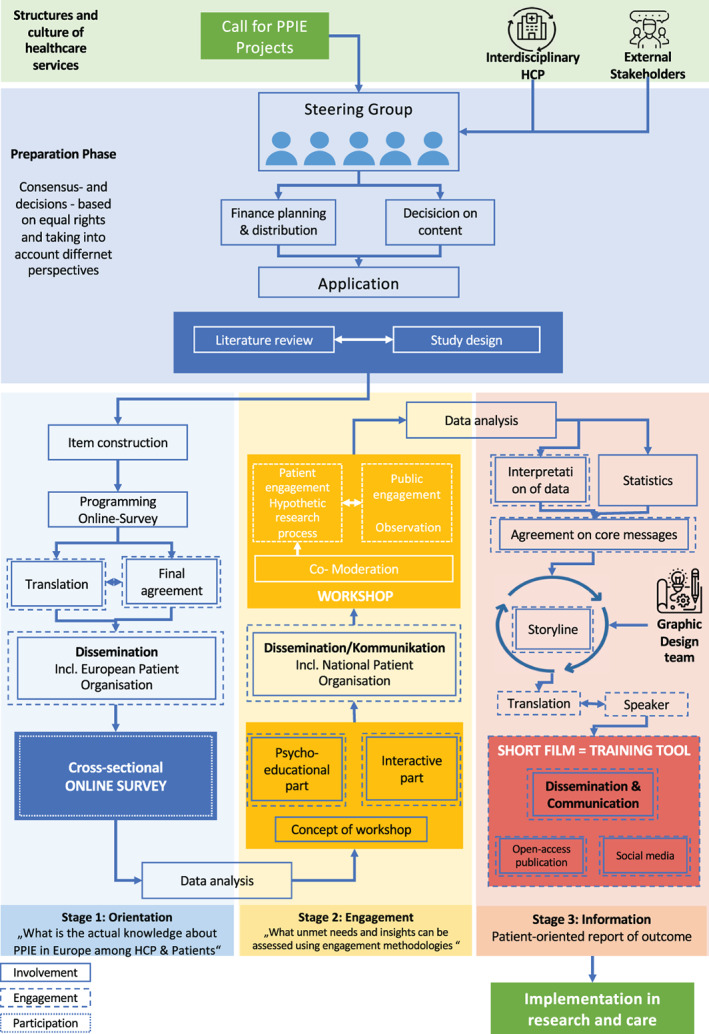
Project framework with indication of PPIE.

An interdisciplinary steering group including psychosocial and medical professionals, as well as patient advocates and patient experts developed a 3‐stage project plan including an orientation, an engagement, and an informational phase. To address both patients and HCP equally and to practice PPIE at every level, a mixed‐method design was chosen. Figure 1 visualizes the process of the present project and indicates where and what type of PPIE was performed and therefore represents the framework of the presented project. Moreover, an exploratory approach was deliberately chosen to enable the use of PPIE methodologies in all steps. Consequently, the research questions, the questionnaire development and administration, and the workshop design can be considered both a method and result of the present research project.

#### 2.1.1 Stage 1: Orientation “Knowledge about PPIE in Europe among HCPs and patients”

In a cross‐sectional design the current knowledge about and establishment of PPIE in the health care sector (HCS) was assessed via a self‐developed questionnaire. This approach should enable the assessment of needs, demands and perceived obstacles for the successful implementation of PPIE throughout Europe.

Based upon prior research on the topic^6^, ^13^, ^15^, ^25^, ^29^ as well as the expertise and experience of the steering group, an online questionnaire was developed with two separate versions aimed at the patient group (individual patients, survivors, family members, patient advocates, patient experts), and at HCPs (e.g., psychosocial professionals, medical doctors, therapists, nurses) respectively. The questionnaires were aimed at adults which is why assistance by a legal guardian was recommended for underaged participants. Both versions included the same basic information on the concept of PPIE and were structured equivalently, assessing the^1^ respondents' demographic information^2^; relation to the health care sector (e.g., illness, profession, or family)^3^; current knowledge about PPIE^4^; ways in which patients are/could be involved in research and development in the HCS^5^; the subjective relevance of research in the HCS^6^; respondents' preceding experience with PPIE; and^7^ challenges and concerns regarding PPIE. The original German version was moreover translated and retranslated by native‐speaking patient experts to English, French, Spanish, and Croatian and pretested among the steering group (HCPs, patient experts, patient advocates). The English version of the questionnaires can be found in Supplement 1. Data security was evaluated by the local ethics commission and a data security statement was included in the questionnaire and had to be accepted by all respondents. The questionnaires were disseminated via the platform SoSci Survey GmbH and distributed throughout the networks of all members of the project team, as well as various social media platforms and by cooperating European patient advocacy groups. Thereupon, the number of actual invitations to the survey can only be estimated (*n* > 1000). The survey was open for participation for 6.5 weeks from September 29th until November 15th 2021.

#### 2.1.2 Stage 2: Engagement “What unmet needs and insights can be assessed using engagement methodologies?”

For the engagement phase, a workshop was designed and piloted on a national level in Vienna to further assess the different stakeholders' knowledge, needs and perspectives and to gather ideas on how and where in the research process PPIE can be established. The workshop concept was based on Polanco et al. (2021)^6^ and was adapted with respect to the results of the previous online survey by a group of *N* = 12 HCPs, patient advocates and patient experts who would also be the moderators during the pilot workshop. The event was aimed at Austrian patients and their families, as well as patient advocates and HCPs. A sign‐up link was distributed via the psycho‐social team of the local pediatric oncology department, a patient organization, as well as through various social media channels. All participants were rewarded with a financial compensation.

Following Polanco et al. example,^6^ the first section of the workshop was filled with short presentations by various professionals to create a common basis for the understanding of scientific research and the concept of PPIE. The lectures provided information on the present research project, the concept of PPIE, the bio‐psycho‐social model^47^ and the consecutive stages of the scientific research process. In open space exercises all members were asked^1^ what they associate with research,^2^ which questions they would like to explore, and^3^ their associations with PPIE. All answers were written down on sticky notes and collected on poster walls.

In a second section, five break‐out groups with two moderators each (1 HCP, 1 patient expert/advocate) were composed by the scientific board (1 clinical psychologist, 1 patient expert) to allow for closer and more effective interaction and more individual contribution within the break‐out groups. There was one children's group with four participants and 4 adult groups which were all composed by a mix of HCPs, individual patients (survivors), families, and patient advocates. With the support and guidance of the moderators, each group then worked through an entire research cycle. Thereby obstacles, ideas for solutions, and visions were discussed for^1^ developing a research question^2^ finding the appropriate methodology, and^3^ analyzing, interpreting, and implementing the results. In a final workshop section, the results of each group were presented in plenary before the achievements of the day were summarized and ideas for the training tool were discussed. The documented input was digitalized and categorized for further analysis. The resulting categories can be found in Table 2.

Throughout the entire workshop day, independent research assistants acted as observers, recording the participants' interactions, thereby focusing on their background and relation to the topic of PPIE. The protocols were predominantly based on the Principles of Successful Patient Involvement by the German Federal Ministry of Education and Research^26^ and included aspects of group dynamics. The evaluation and analysis of the behavioral observations are however not subject to the present research.

#### 2.1.3 Stage 3 – Patient‐oriented report of outcomes

The Third and final stage is the integration of the results of the first two stages following a PPIE rationale. Here, the final goal was to develop a training tool in form of a White‐Board movie, with which the key findings could be communicated to the community including all relevant stakeholders. In a consensus‐based process, the steering group, workshop moderators, and participants used online meetings and email voting to derive key messages and practical implications from all collected data. On that account, the results were statistically analyzed and interpreted by the steering group but also discussed in an explorative approach with a mixed stakeholder group. In cooperation with the communication team and the graphic design team (public engagement), the chosen key messages were then transformed into a story line for the striven White‐Board movie developed in German and English language. The resulting training tool, aimed at all stakeholders, shall be disseminated on social media (YouTube, Facebook, Linked in), organization websites, and used for educational purposes in future workshops and conferences. Additionally, the present open‐access publication was composed by the steering group to serve as a framework and basis for future research.

### 2.2 Sample and participants

To incorporate and visualize all aspects of PPIE (participation, engagement, and involvement), the description of the study population will follow these categories to better define and establish the used terms. Furthermore, the different aspects of PPIE are indicated in all steps of the research process depicted in Figure 1.

#### 2.2.1 Participation – Online survey

Table 1 gives a detailed overview of the survey respondents' sociodemographic data. A total of *n* = 304 participants completed the survey, including *n* = 168 patients/patient‐advocates and *n* = 136 HCPs. Most respondents' countries of origin were Austria, Germany, and Spain with 73% female (female = 221, male = 77, diverse = 1). In the patient group the age was distributed equally. The average age of patients at diagnosis was 7.75 years (*M* = 7.75, *SD* = 8.18).

**TABLE 1:** Sample demographics of online survey participant.

Characteristic	Patients		HCP		Full sample	
	*n*	%	*n*	%	*n*	%
Age						
<14	1	0.6	—	—	1	0.6
14–19	5	3.0	—	—	5	3.0
20–29	39	23.2	—	—	39	23.2
30–39	28	16.7	—	—	28	16.7
40–49	62	36.9	—	—	62	36.9
50–59	29	17.3	—	—	29	17.3
60–69	4	2.4	—	—	4	2.4
70+	0	0.0	—	—	0	0.0
Gender						
Female	120	71.4	101	74.3	221	72.7
Male	44	26.2	33	24.3	77	25.3
Diverse	0	0.0	1	0.7	1	0.3
Not answered	4	2.4	1	0.7	5	1.7
Residence						
Austria	61	36.3	35	25.7	96	31.6
Germany	12	7.1	40	29.4	52	17.1
Spain	45	26.8	19	14.0	64	21.1
Portugal	18	10.7	1	0.7	19	6.2
UK	7	4.2	7	5.1	14	4.6
Italy	4	2.4	8	5.9	12	3.9
Others	24	14.3	25	18.4	45	14.8
Not answered	1	0.6	1	0.7	2	0.7
Nationality						
Austria	61	36.3	34	25.0	95	31.2
Germany	9	5.4	41	30.1	50	16.4
Spain	44	26.2	19	14.0	63	20.7
Portugal	20	11.9	3	2.2	23	7.6
UK	6	3.6	7	5.1	13	4.2
Italy	4	2.4	8	5.9	12	3.9
Others	19	11.3	23	16.9	43	14.1
Not answered	4	2.4	1	0.7	5	1.6
Language						
German	68	40.5	78	57.4	146	48.0
Spanish	42	25.0	20	14.7	62	20.4
Portuguese	19	11.3	3	2.2	22	7.2
English	10	6.0	9	6.6	19	6.2
Italian	5	3.0	8	5.9	13	4.3
Others	18	10.7	16	9.5	34	11.2
Not answered	6	3.6	2	1.5	8	2.6
Language questionnaire						
German	74	44.0	81	59.6	155	51.0
English	38	22.6	30	22.1	68	22.4
Spanish	47	28.0	21	15.4	68	22.4
French	5	3.0	4	2.9	9	3.0
Croatian	4	2.4	0	0	4	1.3
Role patients (multiple selections possible)						
Self‐affected	18	10.7				
Survivor	62	36.9				
Parent	84	50.0				
Sibling	1	0.6				
Patient‐advocate	26	15.5				
Other	17	10.1				
Disease patients						
Oncologic	150	89.3				
Leukemia	45	26.8				
Brain tumor	41	24.4				
Solid tumor	47	28.0				
Endocrinologic	5	3.0				
Hematologic	12	7.1				
Neurologic	7	4.3				
Visceral	1	0.6				
Other	2	1.2				
None	2	1.2				
Therapy method patients						
Operation	97	57.7				
Radiotherapy	79	47.0				
Chemotherapy	146	86.9				
Immunotherapy	17	10.1				
Other	19	11.3				
Profession HCP						
Psychologist			30	22.1		
Social worker			12	8.8		
Psychotherapist			11	8.1		
Pedagogue/educator			7	5.1		
Remedial teacher			3	2.2		
Art therapist			2	1.5		
Music therapist			1	0.7		
Pre‐school teacher			3	2.2		
Medical doctor			58	42.6		
Nurse			13	9.6		
Ergo therapist			2	1.5		
Physiotherapist			1	0.7		
Other			13	9.6		
Area of work HCP						
Pediatrics			122	89.7		
Adult medicine			12	8.8		
Geriatrics			0	0		
Other			11	8.1		
Area of expertise HCP						
Oncology			87	64.0		
Haemato‐oncology			72	52.9		
Neuro‐oncology			35	25.7		
Hematology			26	19.1		
Others			25	18.4		
Setting HCP						
Acute care			93	68.4		
Rehabilitation			11	8.1		
Aftercare			71	52.2		
Research and development			53	39.0		
Care			55	40.4		
Other			9	6.6		
Duration of profession HCP						
0–4 years			37	27.2		
5–9 years			23	16.9		
10–14 years			19	14.0		
15–20 years			20	14.7		
>20 years			37	27.2		
Own role/role of patients						
Individual patient/Career	134	79.8	121	89.0		
Patient advocates	43	25.6	47	34.6		
Patient organization representatives	45	26.8	72	52.9		
Patient experts	42	25.0	39	28.7		

Health care professionals were asked to estimate their work focus regarding medical, psychosocial, nursing, and general care on a continuous spectrum between research^1^ and care (101). On average the estimated focus leaned toward care across all sectors (*M* = 68.57, *SD* = 29.93), with the highest estimates in the psychosocial sector (*M* = 73.91, *SD* = 28.87). In medical care (*M* = 64.91, *SD* = 30.50) and nursing (*M* = 58.28, *SD* = 35.46) the estimated distribution was more balanced yet leaning toward care. Generally, work focus was broadly distributed, spanning from 1 to 101 in all disciplines.

#### 2.2.2 Engagement – Workshop

A total of *n* = 38 people signed up for the workshop via the online link distributed via social media as well as mailing lists and in the personal network of steering group members. One person canceled on the day of the event, and one individual patient was excluded due to considerable disease‐related difficulties in social interactions. The final group of *n* = 36 participants was comprised by *n* = 5 HCPs (4 clinical psychologists; 1 cancer researcher); *n* = 11 pediatric cancer survivors; *n* = 9 parents and *n* = 4 other relatives of childhood cancer patients; *n* = 1 patient advocate; and *n* = 6 students. The mean age of adult participants was *m* = 32.69 (*SD* = 9.48) and the four adult groups were comprised by 7–9 people equally distributing the stakeholders. The children's' group included 2 survivors, one sibling and one member of the public with a mean age of *m* = 7.25 (*SD* = 0.96).

#### 2.2.3 Involvement – All stages

At the beginning of this project, the steering group consisted of *n* = 5 members including three HCPs (scientific practitioners and scientific physicians), one patient expert, and one patient advocate. However, this group evolved throughout the project leading to a total number of *n* = 12 workshop moderators, who were grouped into dual moderation teams with one HCP and one patient expert or patient advocate each. In the third and final consensus‐phase in Stage 3, the working group included *n* = 17 people including the original steering group, the moderators, individual patients, patient advocates and a graphic design team (public engagement).

### 2.3 Data analysis

All quantitative and qualitative analyses were performed by the scientific board constituted by a clinical psychologist and a patient expert, with the support of project assistants.

#### 2.3.1 Quantitative analysis

For this exploratory study, descriptive analysis was used for the questionnaire as well as the categorical data collected during the workshop. A significance level of *α* = .05 was applied and the software package R was utilized for all calculations.^48^ All graphs were compiled with the package “ggplot2.”^49^ Due to the large group sizes independent group t‐tests were used to compare the mean responses on continuous scales. Conventional Cohen's *d* was used to describe effect sizes (*d* ≥ 0.2 = small; *d* ≥ 0.5 = medium; *d* ≥ 0.8 = large effect). A Multi‐Way ANOVA was used to investigate group differences in the continuous distribution of responses. To analyze group differences for the frequency of multiple‐choice answers, Chi‐square test of independence was used, whereby effect sizes were described using Cramer's V. If test requirements were not met due to low case numbers, Fisher's exact test was applied instead.

#### 2.3.2 Analysis of qualitative data

First, the qualitative data collected via the open‐end questions in the questionnaires were reviewed for completion and the French and Croatian data was translated into German via the free online translation service deepL for categorization. The data collected on poster walls during the workshop was transcribed, digitalized, and categorized. Second, the scientific board grouped the qualitative data collected during the survey as well as the workshop into classifications according to the predominant theme of the statement, resulting in different divisions for each of the assessed dimensions. In a third step, pre‐existing categories derived from the literature^13^, ^15^, ^47^ were used and applied to the corresponding data to align it with the quantitative data in the questionnaire. The resulting final set of categorizations used for quantitative analysis can be found in Table 2. The open‐end descriptions of the three types of PPIE in the questionnaire were categorized into 4 groups regarding correctness, namely correct per definition, incorrect per definition, no response, evaluation instead of definition.

**TABLE 2:** Categories of open responses to survey questions for quantitative analysis.

Associations with research	Possibilities for PPIE	Possibilities for PPIE	Challenges	Possibilities for Involvement workshop	Associations with PPIE workshop
Defining the research question	Participation	As study participant	Barriers (social, language, emotional, age)	Defining research question	Participation
Research design and planning	Engagement	Answering questionnaires	Discrepancies in different stakeholders aim	Research design and planning	Involvement
Data collection	Involvement	Reviewing patient‐brochures	Structure of health care system	Data collection	Engagement
Data analysis	Other	Reviewing patient information and informed consent papers	Quality of Life, well‐being and after care	Data analysis	Other
Communication of results		Reviewing submissions to ethics committees	Shortcomings (knowledge, information, understanding)	Communication of results	
Implementation of results		Co‐planning design and equipment of health care facilities (e.g., when a new hospital or ward is being built etc.)	Objectivity	Implementation of results	
Study participation		User ratings of research results (e.g., of services or programs for patients and their families, new treatment methods etc.)	Resources	Study participation	
Association with PPIE		Research funding (e.g., raise funds/donations for research projects)	Collaboration	Other	
		Setting research priorities	No response		
		Defining research questions	Other		
		Development of patient brochures			
		Development of patient information and informed consent papers			
		Development of research‐/project proposals			
		Development of ethics proposals			
		Development of treatment‐protocols			
		Development of study reports/data analysis			
		Post‐study communication			
		Contribution to publications			
		Dissemination of research results to patient community			
		As members of steering committees			
		As members of data‐safety/monitoring committees			
		Participation in investigator‐meetings			
		Not at all			
		Other			

## 3 RESULTS

### 3.1 Stage 1: Orientation “Knowledge about PPIE in Europe among HCP and patients”

#### 3.1.1 Associations with research and development in health care

All participants were asked to state their associations with research and development in the health care sector. Statements from both groups showed significant differences regarding the category they counted toward (*X* ^2^ (6, *N* = 304) = 59.05, *p* < .001). While HCPs mainly mentioned concepts related to research projects and the research environment, patients mainly stated associations with research results.

#### 3.1.2 Expertise in PPIE (“That's how well I know the subject of PPIE”)

**FIGURE 2:**
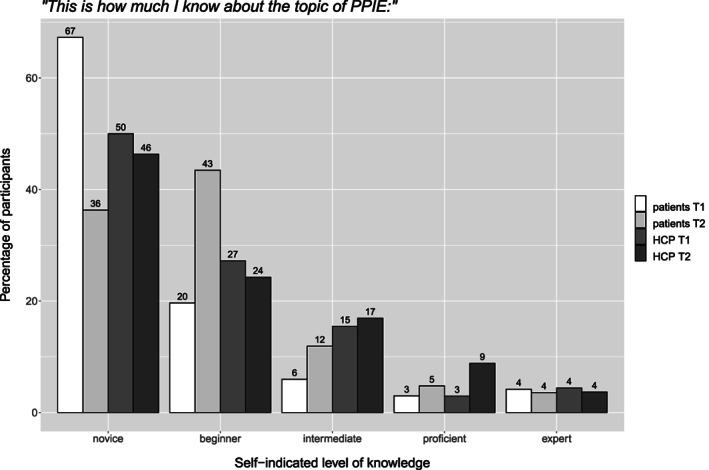
Self‐assessment at beginning (lighter shade) and at the end of the survey (darker shade).

On a scale from beginner^1^ to expert^5^ the participants' subjective expertise in PPIE at the beginning and end of the survey was rather low in both groups (patients/patient‐advocates: *M* = 1.57, *SD* = 1.02; HCP: *M* = 1.85; *SD* = 1.07). Still, HCPs assessed themselves as significantly more informed than did patients (*t*(302) = −2.27, *p* = .023, effect size: *d* = 0.26). In both groups, participants rated their own expertise significantly higher in the second, compared to the first assessment (patients: *M* = 1.89, *SD* = 1.03, *t*(334) = −2.82, *p* = .004, *d* = 0.31; HCPs: *M* = 2.21, *SD* = 1.03, *t*(270) = −2.88, *p* = .004, *d* = 0.35). See Figure 2 for details.

#### 3.1.3 Knowledge about PPIE terminology (“This is how well I know the following terms in the context of PPIE”)

**FIGURE 3:**
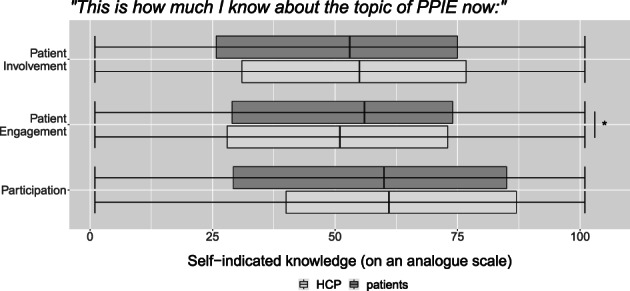
Subjective knowledge on a continuous scale from Do not know at all (1) to Know it very well (101), significant differences are marked with *.

As visualized by Figure 3, knowledge was spread across all levels for each term, although patients indicated lower knowledge of each concept than HCPs. With respect to the term “participation” the self‐assessed knowledge was significantly higher in HCPs (*t*(270) = −3.07, *p* = .002, *d* = 0.37).

When describing these terms in their own words, the patient group correctly assigned the concepts in 20.36% for patient involvement, 20.96% for participation, and 8.98% for patient engagement. For the HCP‐group, the percentage of correct description was as follows: patient involvement = 31.34%, participation = 26.87%, patient engagement = 17.91%. Generally, there were high rates of missing responses.

#### 3.1.4 Possibilities for involvement in research (“When you think about research and development: Where/How can patients be involved?”)

**FIGURE 4:**
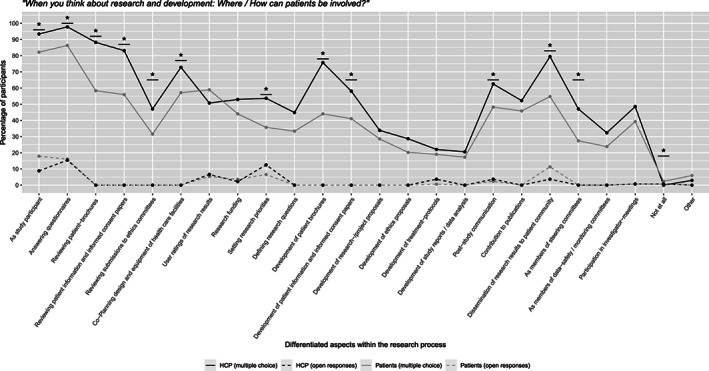
Agreement to “Where/How can patients be involved?” in patients and HCPs.

In a multiple‐choice format most participants indicated that patients can be involved as study participants, by answering questionnaires and by reviewing patient‐brochures. The two groups also agreed that the development of patient‐brochures and the dissemination of research results to patients could be a major part of patient involvement. Generally, HCPs had higher agreement throughout the categories, with significant differences in 12 out of 22 research steps, while the patient groups significantly more often stated that patients could not be involved at all. In an open response format participants in both groups named numerous options, however, most of these ideas could not be counted toward a specific research step. The responses which qualified to be categorized predominantly fell under the classifications answering questionnaires, setting research priorities and the dissemination of research results to the patient community. For more details see Figure 4.

#### 3.1.5 Personal involvement in medical, psycho‐social, and nursing research

When asked for their experience with involvement in different research areas, around half of the patients (49.4%) claimed to never have been involved in research at all, compared to 13.2% of HCPs never having involved patients (*χ* ^2^(1, *N* = 304) = 42.70, *p* < .001, Cramer's *V* = 0.38). HCPs indicated that they had involved patients significantly more in medical research (*χ* ^2^(1, *N* = 304) = 6.44, *p* = .011, Cramer's *V* = 0.15) and psycho‐social research (*χ* ^2^(1, *N* = 304) = 26.01, *p* < .001, Cramer's *V* = 0.29), while in nursing research there was no significant difference.

#### 3.1.6 Personal involvement in research and development

**FIGURE 5:**
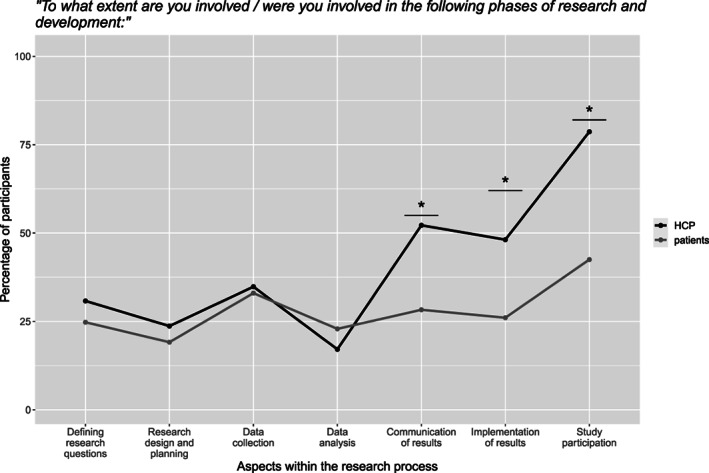
Amount of involvement of patients/by HCPs.

On a continuous scale [1;101] all participants indicated how much they were involved or actively involved others in different areas of research. Generally, the involvement was low, especially according to patients' estimation, where the mean score did not exceed 39 for any of the research areas. As illustrated in Figure 5, patients and HCPs did not differ in their estimated involvement in earlier stages of research (defining the research question, research design/planning, data collection, data analysis) with both groups indicating low levels of involvement. In contrast in the later phases, HCPs estimated the extent to which they involved patients to be significantly higher than patients estimated their own involvement [communication of results (*t*(262) = −4.30, *p* < .001, *d* = 0.54); implementation of results (*t*(266) = −3.88, *p* < .001, *d* = 0.48); study participation (*t*(265) = −6.85, *p* < .001, *d* = 0.85)].

#### 3.1.7 Challenges (“Which challenges or concerns come to your mind when you think about involvement of patients in research and development according to the concept of PPIE?”)

**FIGURE 6:**
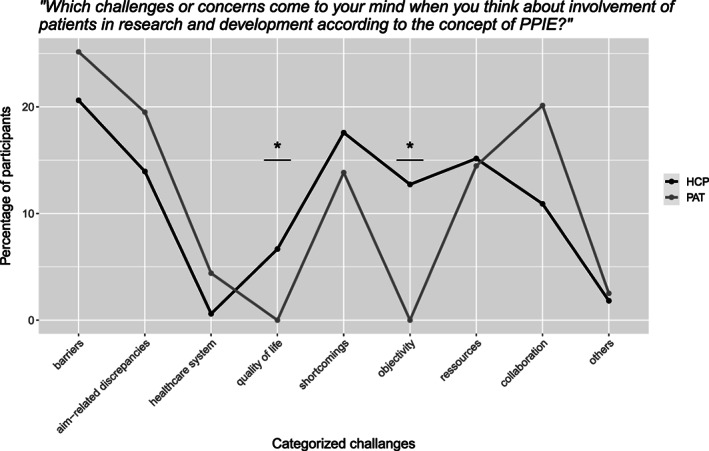
Categories of answers concerning challenges of PPIE.

In the open‐end question both groups named a high number of potential challenges. Categorization into eight listings showed that both groups cited challenges in form of barriers, aim‐related discrepancies, general shortcomings, and missing resources and collaboration. HCPs significantly more often mentioned challenges regarding patients' objectivity (*p* < .001, Fisher's exact test) and quality of life (*p* < .001, Fisher's exact test). Figure 6 visualizes the relative number of answers per category.

### 3.2 Stage 2: Engagement “What unmet needs and insights can be assessed using engagement methodologies”

#### 3.2.1 Open space

The resulting *n* = 67 associations considering “research” in general were recorded into the categories “research vocabulary” (70%) (e.g., “enables progress and broadens horizon,” “generating new knowledge,” and “development”), “PPIE topics” (4%) (“recognizing needs,” and “better explanations – no specialist terms”), and “valuations” (25%) (“necessity,” “curiosity,” and “independence”). When asked for research questions the participants would like to investigate, a total of *n* = 44 topics was named, which due to the great variety and heterogeneity could not be categorized. Some questions were very specific to one research area such as medication, patient well‐being (“The impact of information on patient's psychological well‐being”), school reintegration or PPIE (“Which adaptions does the health care system need in order for PPIE to be implemented?”). Others were broader and less specific to one research area for example, “How can we connect different worlds?” or “Why do we speak so little about research?”

When asked for “associations with PPIE,” a total of *n* = 43 statements was produced whereof 79% were related to PPIE (e.g., “everybody is an expert!”*, “Communication is the key!”*, “practical integration of different perspectives”*, “teamwork makes the dream work,” and “interdisciplinary implementation”).^1^

#### 3.2.2 Breakout sessions

For each of the three discussed research stages, participants were asked to name potential challenges, possible solutions, visions, and conclusions. The challenges were categorized into HCS‐related (e.g., “resources,” “financing,” “medical and nursing education”), patient‐related (e.g., “language barriers,” “knowledge,” “physical distances”) and other challenges (e.g., “Corona,” “safety”). The ideas for solutions were recorded into solutions provided by traditional research methods (e.g., “mobile psychology,” “interpreting”), PPIE‐related solutions (e.g., “exchange,” “involving everybody early on,” “research workshops for lays”) and other ideas (e.g., “democratic approach,” “using smileys”).

In the children's group less written data was generated since the workshop was more interactive with many creative activities such as drawing their own superpowers and associations. The behavioral observation, however, revealed a high level of interaction, with the two survivors and the sibling communicating extensively about the time during treatment, what challenges they faced and what they would have liked to be different.

A detailed overview of the different categories and numbers for the workshop data can be found in Table 3.

**TABLE 3:** Categorized workshop data.

Category	Total number	%
Associations with research	67	
Research vocabulary	47	70
PPIE topics	3	4
Valuations	17	25
Associations with PPIE	43	
PPIE	34	79
Not PPIE	9	21
Suggestions for research questions	44	
From need to research question		
Challenges	54	
Related to HCS	37	69
Related to patients	4	7
Other	13	24
Solutions	36	
Traditional research	9	25
PPIE	11	31
Other	16	44
Visions	24	
Conclusions	8	
From research question to methodology		
Challenges	14	
Related to HCS	8	57
Related to patients	4	29
Other	2	14
Solutions	44	
Traditional research	13	30
PPIE	12	27
Other	19	43
Visions	14	
Conclusions	9	
From methodology to result		
Challenges	10	
Related to HCS	6	60
Related to patients	2	20
Other	2	20
Solutions	16	
Traditional research	8	50
PPIE	4	25
Other	16	25
Visions	16	
Conclusions	13	
Children's group		
Superpowers	8	
Associations with health	2	
Associations with illness	12	

### 3.3 Stage 3: Patient‐oriented report of outcomes

**FIGURE 7:**
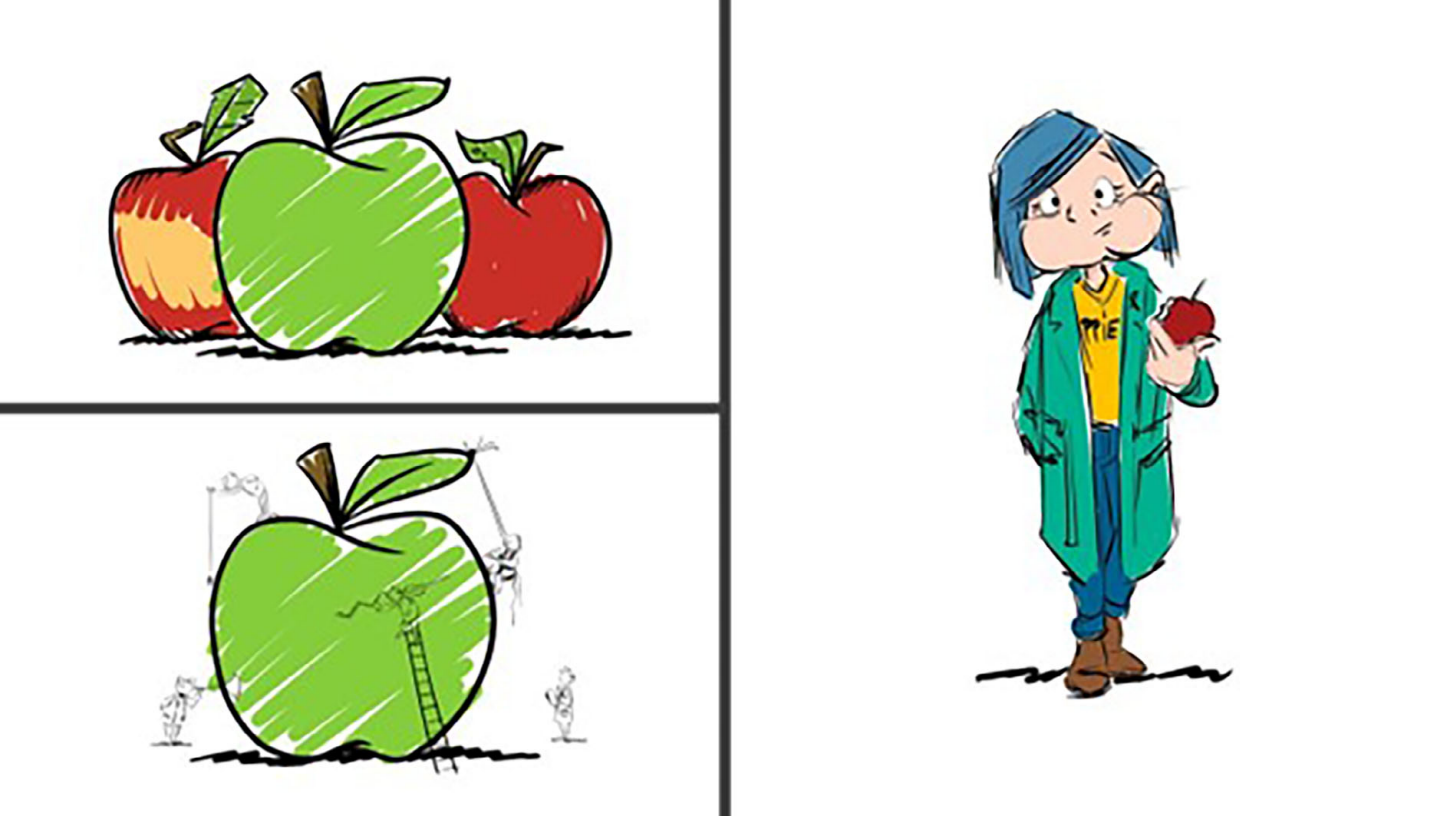
Film scene of patient advocate “Claire” describing PPIE with the metaphor of the patient perspective who had to bite into the sour apple and therefore shares a unique expertise.

The collaborative production merging the outcomes of stages 1 and 2 resulted in a selection of metaphors and images for one unifying White‐Board movie, equally directed at all stakeholders and HCPs. This tool aims at explaining the basics PPIE terminology, the various roles patients and the public can adopt, and the various possibilities its implementation. Figure 7 represents an exemplary scene of the movie depicting a metaphor used to describe the value of the patient perspective in the sense of PPIE. The tool was published on YouTube and disseminated through various social media platforms. Furthermore, it was presented on congresses and during workshops and used for educational purposes.

**Link to training tool:** “Good to know! That's PPIE (English)” https://www.youtube.com/watch?v=HHITKZ5tZcY&ab_channel=COPEGroup.^50^

## 4 DISCUSSION

The present project aimed at exploring, creating, using, and evaluating various renown as well as new PPIE methods, which is why PPIE can not only be considered the research subject but also the methodology used to investigate it. A 3‐stage project plan, implementing all aspects of PPIE (participation, engagement, and involvement) was used to first generate knowledge about the current establishment of PPIE before using involvement of patients and the public to integrate the results into a training tool capable of conveying the key findings to the community including all relevant stakeholders in pediatric oncology. The various methods used in the study design can already be considered a major result regarding successful PPIE and thereby represent a first step toward effectively establishing PPIE in scientific research in pediatric oncology. The present project serves as an example for how all relevant stakeholders can be involved in project design and study prioritization, since the steering group of the entire project was composed by HCPs as well as patient experts and advocates. The cooperation allowed for an integration of all perspectives throughout the entire research process, including the construction, distribution, and evaluation of the online survey; the design and realization of the workshop; the construction and dissemination of the training tool; and the authoring of the final report. The derived workflow (Figure 1) can therefore serve as framework and practical guidance for future projects aiming at implementing PPIE, since it highlights necessary elements of participation, engagement, and involvement and allows for the individual adaptation to the specific circumstances of future research.

Second, the online survey in Stage 1 (Orientation) allowed for detailed insights into the status quo of PPIE in pediatric oncology across Europe to understand knowledge, approaches, and attitude of both HCP and patients equally. In line with prior research looking into the currently practiced degree of involvement,^9^, ^18^, ^21^ extensive deficits in the establishment of PPIE came to light. Thereby significantly more patients stated to never have been involved in any type of scientific research while HCPs claimed to involve patients significantly more in the later research steps. This lack of awareness among the patient group about having been participating in studies indicates deficient communication between stakeholders, suggesting that patients do not always understand the informed consent forms and are unaware of the fact that their treatment in the clinic makes them a vital part of research.^33^, ^35^ Conversely, all stakeholders agreed on low levels of involvement in the early stages of research. These findings contrast with the manifold options and wishes for PPIE named by the participants as well as a with a high percentage of the multi‐facetted possibilities for PPIE (as suggested by Hunter et al.) the participants considered feasible.^15^ In summary, these results can serve as a basis for the development of novel techniques for implementing PPIE as they reflect an unbalanced implementation of the different types of PPIE as well as the need for more involvement throughout the entire research process. Thereby new strategies should especially focus on the involvement in early phases of research to elevate the now predominantly passive participation to the level of actively shaping research questions to genuinely emancipate the opinion of the patient group and to avoid tokenism. Furthermore, authors should clearly indicate PPIE as an integral part of research to ensure a common understanding and clear communication of where and when PPIE is practiced.^21^, ^30^, ^51^, ^52^ Finally, it is hence essential to increase the transparency of and education on current research for the public, as well as to set compulsory standards for research to involve patients into the entire process. Thereby a familiarization of all stakeholders with the manifold options of involvement could be achieved and the quality of PPIE could be warranted by making sure it is not only practiced where it can be implemented easily, but instead where it is most relevant for the emancipation of the patients' interests and needs.

The presumed lack of official establishment of PPIE was further affirmed by a low expertise on PPIE among survey participants,^12^ although it already became clear during item construction where English terms needed to be used in the German, Spanish, French, and Croatian translations of the questionnaire. This highlights that although the importance of public involvement is for example, stressed in the *Best Practice Guide for Research Integrity and Ethics* by the Austrian Federal Ministry of Education, Science and Research (BMBWF),^53^ legally binding rules for involvement are still lacking in many European countries.^6^ Furthermore, the survey revealed a low general level of knowledge on the specific PPIE terminology with few participants being able to correctly describe participation, involvement, or engagement. In addition to the general ignorance toward PPIE, another reason for the lacking specific knowledge could be the considerable variance in research terminology,^6^ which is why for the present project the concepts participation, engagement, and involvement as defined by the NIHR were used.^13^ Similarly, most options and wishes for involvement named in an open response format could not be categorized as any type of PPIE. This unclarity of language and terminology stresses the deficit of information on the topic while also highlighting the risk of misunderstandings in the communication about PPIE. Therefore, effective engagement such as shown in Stage 2 (Engagement) of the present project is necessary where after one workshop day participants were already able to correctly use PPIE vocabulary and to define clear needs in their associations with PPIE. To further foster effective communication about and valid research on PPIE, a clear consensus on this matter and education on its definition, as well as uniform terminology is essential.^15^, ^37^ All things considered these findings strengthen the concept of engagement as approached in Stage 2 (Engagement) of this project.

In line with prior research on obstacles to the effective implementation of PPIE,^5^, ^7^, ^21^, ^29^, ^30^ the most relevant challenges indicated by both groups were differences in prioritization and values, and a lack of understanding and unsuccessful communication, not only between HCPs and the patient group, but also among the different areas of the health care sector (medicine, psychology, social work, nursing). Subjective reasons for these barriers included the inaccessibility of information due to a deficit in lay summaries and patient engagement and diverging priorities of the different stakeholders. Discrepancies in perspectives are further indicated by the fact that two barriers were exclusively named by HCPs, namely a low quality of life burdening the patients, as well as a lack of objectivity possible for members of the patient group. This suggests that HCPs assume burdens and inaptitude of patients to be involved, where the patient group perceives none, and that the expertise brought about by the patients' personal experience is not regarded by HCPs. However, as summarized by Price et al.,^5^ it is precisely this regard and acknowledgement of the patients' perspective in combination with a clear definition of roles and methods that forms the basis for a sustainable establishment of PPIE as an integral part of all research in the health care sector.

In this context, the workshop in Stage 2 (Engagement) proved to be a helpful method to address exactly these issues of ineffective communication and lacking collaboration serving both as a means of gathering ideas for successful PPIE, as well as a way of putting PPIE into practice by bringing HCPs and the patient group together to evaluate the opportunities and challenges for patient involvement during the various stages of research. In addition, the participatory online questionnaire and the workshop brought distinct aspects to light. The analysis of data collected during the workshop in Stage 2 (Engagement) showed, that an open discussion and cooperation on eye level between all stakeholders allowed for a great variety of complex, heterogeneous potential challenges to be counterbalanced by a large number of creative solutions. Consequently, engagement could not only inform participants, but also allowed producing knowledge and new creative research ideas. These findings provide a basis for the development of tools to systematically record and communicate these solutions, making them accessible for implementation groundwork as a basis for research and care better tailored to patients' needs. The need for such new assessment methods is especially pronounced regarding the perspective of children, which to date cannot be meaningfully recorded and therefore also not incorporated into research and development. Furthermore, future research needs to work on appropriate communication methods, soft skills and a common vocabulary for HCPs and the patient group to foster the openness as well as close collaboration and integration of perspectives that so ineffably enriched the findings of the present project.

In the final Stage 3: (Patient‐oriented report of outcomes), the results of the preceding steps were collaboratively integrated into the training tool to disseminate the gathered information to the community. During the survey and workshop, it became evident that there first needs to be an increase in knowledge and awareness for the topic before practical indications and guidelines for PPIE can be understood and sustainable official establishment of PPIE can be achieved. Therefore, the training tool in form of a White‐Board movie was designed to educate on the concept rather than ways of achieving PPIE, by conveying the key‐messages the extended working group agreed upon. It was designed to be as inclusive as possible, directed toward all different stakeholder groups, and there was an emphasis on the establishment of PPIE as joint responsibility of both parties. This required a focus on the respectful cooperation of all stakeholders as an invaluable basis for effective and successful PPIE that is beneficial to all parties. Although this main result of the present project differs from traditional research results, the form of a White‐Board movie was deliberately chosen to follow the rational of PPIE, making the results as approachable and accessible as possible for all relevant stakeholders. It can be assumed that the application of PPIE requires breaking down or at least adapting current research methods. Furthermore, inclusion of patients allows for new perspectives and insights that would not have been uncovered with established methods. However, it should be noted that more concrete methods for PPIE will be needed in the future to standardize and quantify these processes (which people, clarification of roles, shared decisions, interpretation of results, …). In addition, the assumption of perspectives and the willingness to enter the world of the other group are prerequisite for a shared research process to reach the same goal of improving care and research equally.

## 5 LIMITATIONS AND FUTURE DIRECTIONS

Following the rationale of implementation sciences, it cannot clearly be distinguished, whether PPIE is the subject or method of the present project which adds considerable complexity to its description. While this limited clarity and loosely defined research questions can be considered a limitation in traditional research perspectives, the openness can also be seen as a new approach and perspective in research and therefore as a strength in the context of PPIE, since room was left for the integration of different perspectives and additional aims. The aim of the survey was to reach as many respondents as possible through the available networks, yet translations were limited to the most widely spread European languages English, German, French, Spanish, and Croatian due to the restricted availability of translators. Nationalities of respondents were clustered in the countries where contact with active collaborators was possible. Furthermore, a representation bias needs to be considered when interpreting the results, since the distribution through patient organizations likely caused people already involved in and acquainted with the topic to be overrepresented. Additionally, there is a clear tendency toward female respondents and a more balanced sample is necessary to investigate the influences of sociodemographic characteristics such as socioeconomic status and education on the resulting response patterns. Future research is necessary to achieve a more homogeneously distributed European sample using validated translations to more languages which we hope to be facilitated by a general increase in knowledge and awareness about the topic. For the analysis of qualitative data, the categories were defined a priori which allows for less generation of knowledge than a more inductive, Grounded‐Theory approach might do. In this project an exploratory approach was chosen to get impartial input on the posed research questions. Yet for future projects, more valid measures for the qualitative responses and operationalization methods of research processes and group activities are necessary to allow for better categorization and their consequent analysis to conduct a systematic evaluation of the workshop. This is especially necessary while working with children to ensure that their perspectives are appropriately recorded and incorporated. Finally, the survey and workshop pilot lay the basis for a follow‐up engagement project named “Junior Research Academy,” where a more concrete directive for effective engagement and involvement in the research process in pediatric oncological research shall be developed.

## 6 CONCLUSION

This study on present‐day practice of PPIE in pediatric oncology across Europe and relevant stakeholders highlighted the need for a clear definition and a collective understanding of the concept of PPIE as a basis for its sustainable and comprehensive implementation. To counter the lacking knowledge and awareness, a training tool was created such that it would provide basic knowledge on the concept as well as on the possibilities for involvement and the potential challenges to be faced. Thereby, one version was created using public and patient involvement to educate HCPs and the patient group and public equally and to further foster a concurring understanding and a shared language as a prerequisite for effective communication on the topic. This tool is freely available on social media and has been used for educative purposes in workshops and scientific congresses. Finally, the framework of the present study serves as an example for how future studies can implement PPIE in all steps throughout a multi‐level research process which would represent a first step toward a more inclusive and emancipating scientific research culture in the health care sector.

## AUTHOR CONTRIBUTIONS

**Liesa Josephine Weiler‐Wichtl:** Conceptualization (equal); formal analysis (equal); funding acquisition (equal); investigation (lead); methodology (lead); project administration (equal); supervision (lead); visualization (equal); writing – review and editing (equal). **Ulrike Leiss:** Conceptualization (equal); funding acquisition (equal); investigation (equal); methodology (equal); supervision (equal); writing – review and editing (equal). **Johannes Gojo:** Conceptualization (equal); investigation (equal). **Anita Kienesberger:** Conceptualization (equal); funding acquisition (equal); investigation (equal); supervision (equal). **Rita Hansl:** Data curation (supporting); formal analysis (supporting); investigation (supporting); visualization (supporting); writing – original draft (equal). **Maximilian Hopfgartner:** Data curation (equal); formal analysis (equal); visualization (equal); writing – original draft (equal). **Carina Schneider:** Conceptualization (equal); data curation (equal); funding acquisition (equal); investigation (equal); methodology (equal); project administration (equal); supervision (equal); writing – review and editing (equal).

## CONFLICT OF INTEREST STATEMENT

The authors have stated explicitly that there are no conflicts of interest in connection with this article.

## ETHICS STATEMENT

In consultation with the Ethics Committee of the Medical University of Vienna no ethical approval was needed for the data collection, workshop, and data analysis. A data protection statement was provided by the ethics committee and all participants gave written consent to the anonymous use of the provided data as well as the recording and analysis of written notes during the workshop.

## Supporting information


**Table S1:** Online Survey Questionnaire (English).

## Data Availability

The data that support the findings of this study are available from the corresponding author upon reasonable request.

## Endnote

^1^
* = concepts named repeatedly.
