# Supplementary material for: Good to know – This is PPIE! Development of a training tool for public and patient involvement and engagement in pediatric oncological research
Source: Cancer Rep (Hoboken). 2023 May 11;6(6):e1835. doi: 10.1002/cnr2.1835 (PMC10242658; doi:10.1002/cnr2.1835)
Supplement: Supplementary file 1 — Table S1: Online Survey Questionnaire (English). [file CNR2-6-e1835-s001.pdf]

Table S1: Online Survey Questionnaire (English)

<table>
<tr><th>Health care professional version</th><th>Patient group version</th></tr>
<tr><td colspan="2">Page 0: Language selection</td></tr>
<tr><td colspan="2">Deutsch English Español Français
Hrvatski</td></tr>
<tr><td colspan="2">Page 1: Introduction</td></tr>
<tr><td colspan="2">Dear participant!

We kindly invite you to participate in our survey for the project "Nothing about us without us! PPIE in paediatric oncology? That's how it works!".

PPIE is short for Patient and Public Involvement and Engagement in healthcare research and development (you can find more details in the info box).

Within our 1-year-long project "Nothing about us without us! PPIE in paediatric oncology? That's how it works!" we aim to develop a training program and a short video to increase awareness for PPIE in the paediatric oncological community and facilitate its implementation. Both the training program as well as the awareness-video will address patients and their families as well as health care professionals and patient-organizations.

To be able to tailor the tools towards the needs of each targeted groups, we first want to evaluate the current state of knowledge and awareness about the concept of PPIE and about how extensively the different groups are currently involved in research and development. Therefore, we are carrying out the present survey targeted at patients and their families as well as health care professionals and patient organizations.

How does the survey work?

To ensure that the data you provide us with can be used for our project, you will find a statement of informed consent on the following page which needs to be acknowledged. Thereby we guarantee that all collected data is anonymous and will exclusively be used for research purposes in the realm of our project.

After that are our questions. It will take about 10-15 minutes to answer the survey. You do not need any prior knowledge in order to take part, and there are no "incorrect" answers!

Thank you for taking part in our survey!

Ready? Then let's get started! :)

Check the following boxes for more information:</td></tr>
</table>

What initated this project?

It is our aim to raise awareness for PPIE among the childhood cancer community and to implement the concept in the research culture – because we know that the active involvement and engagement of patients is an enormous asset for research, and that the results have direct impact on the every-day life of patients. The Ludwig-Boltzmann-Society thinks that too and opened a grant to support projects which explicitly foster the implementation of PPIE in research. Our project team – next to 11 other projects – was awarded the project grant and now we have 1 year to make our project **„Nothing about us without us! PPIE in paediatric oncology? That's how it works!"** become

Who can participate in the survey?

Since our team is working in pediatric oncology, the focus of this survey is on patients with pediatric-oncological diseases and their families as well as health care professionals in this area (psychosocial professionals, doctors, nurses, researchers etc.). However, we are more than happy about every person who is willing to answer our questions regarding this important topic, which is relevant to the various disease areas. That is why we made sure that all our questions can be answered regardless of the disease area.

PPIE? Never heard of it! What's that supposed to be?

PPIE is short for „Patient and Public Involvement and Engagement" in research and development, so for instance the active involvement of patients or members of the public when it comes to research regarding certain diseases or the development of programs in the health sector. The overarching goal of PPIE is to develop programs and research projects with a high every-day relevance for patients and the public. This aims to ensure that the respective research and its outcomes really do reflect the needs of the patients.

Who are „we"?

We are a multi-professional team consisting of psychologists, pediatric oncologists, nurses, and patient-experts from the [redacted]

Page 2: Screening for correct questionnaire selection

I participate in this survey as:

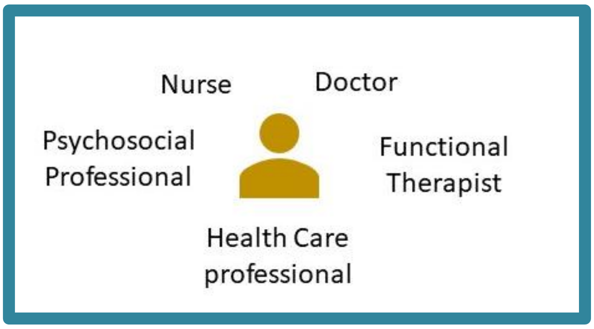

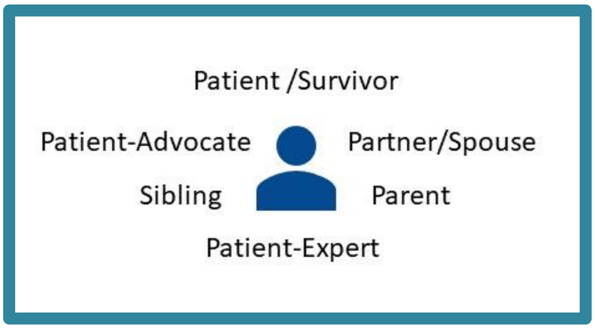

Page 3: Informed consent

<u>Privacy Statement</u>

As part of the survey project "Nothing about us without us! PPIE in paediatric oncology? That's how it works!", the Medical University of Vienna (MedUni Vienna), as the legal data protection officer, is evaluating your responses.

The company SoSci Survey GmbH (Marianne-Brandt-Str. 29, 80807 Munich, Germany), with which MedUni Vienna has concluded an agreement on commissioned processing in accordance with Article 28 of the Data Protection Regulation, will provide you with the online questionnaire. The privacy policy of SoSci Survey can be found here: https://www.soscisurvey.de/de/data-protection. Both the company headquarters and the server are located in Germany, which means that the strict data protection regulations of the General Data Protection Regulation also apply to SoSci Survey GmbH.

Within the scope of this survey, personal data about you will be collected and processed.

A fundamental distinction must be made between:

1) those personal data by which a person is directly identifiable (e.g. name, date of birth, address, national insurance number, picture recordings...),
2) pseudonymised personal data, i.e. data in which all information that allows direct conclusions to be drawn about the specific person is either removed, replaced by a code (e.g. a number) or (e.g. in the case of photographs) made unrecognisable. However, despite compliance with these measures, it cannot be completely ruled out that re-identification may occur.
3) anonymised data, for which a traceability to the specific person can be ruled out.

Within the framework of this survey, we process data such as gender, age group, nationality, mother tongue, occupational group, diagnosis group on the basis of your consent, which can be revoked at any time, and which you express by completing and submitting the online survey.

In the context of this questionnaire survey, no data will be passed on outside the EU or EEA (third country).

Your consent forms the legal basis for the processing of your personal data. You can revoke your consent at any time without giving reasons. The revocation of consent does not affect the lawfulness of the processing carried out on the basis of the consent until the revocation.

The data disclosed by completing the questionnaire will be stored exclusively on a password-protected workstation at MedUni Vienna for a maximum period of 10 years from the end of the project.

In principle, you have the rights to information, correction, deletion, restriction of processing, data transferability and objection. To exercise your rights, please contact liesa.weiler@meduniwien.ac.at and/or the MedUni Vienna data protection officer at datenschutz@meduniwien.ac.at.

If you are of the opinion that the processing of your data violates data protection law or that your data protection rights have been violated in any other way, you can lodge a complaint with the supervisory authority (https://www.dsb.gv.at/).

Declaration of Consent

- I have read and taken note of the privacy policy. (forced selection)

Page 4: Demographic data

My sex:

- female
- male
- intersex
- I prefer not to say

I currently live in:

(Single choice selection of countries)

My nationality:

(Single choice selection of countries)

My age:

- <14
- 14-19
- 20-29
- 30-39
- 40-49
- 50-59
- 60-69
- 70+

My sex:

- female
- male
- intersex

**My mother tongue:**<br>(Single choice selection of languages)	• I prefer not to say<br><br>**I currently live in:**<br>(Single choice selection of countries)<br><br>**My nationality:**<br>(Single choice selection of countries)<br><br>**My mother tongue:**<br>(Single choice selection of languages)
**Page 5: Anamnesis**	
**I am a...**<br>(Multiple choice)<br>• (clinical) Psychologist<br>• (clinical) social worker<br>• Psycho therapist<br>• Pedagogue<br>• Remedial teacher<br>• Art therapist<br>• Music therapist<br>• Educator/ Kindergarden teacher<br>• Doctor<br>• Nurse<br>• Occupational therapist<br>• Physio therapist<br>• Logo therapist<br>• Functional therapist<br>• Other option: _______<br><br>**My discipline:**<br>(Multiple choice)<br>• Oncology<br>• Haematooncology<br>• Neuro-oncology<br>• Hematology<br>• Other:<br>○ General Surgery<br>○ Anaesthesiology and Intensive Care Medicine<br>○ Angiology<br>○ Ophthalmology<br>○ Dermatology and Venereology<br>○ Gynaecology and obstetrics	**I am a...**<br>(Multiple choice)<br>• Patient<br>• Childhood/Adolescent/Young adult Cancer survivor<br>• Parent of a child/adolescent/young adult who is/was in treatment for cancer<br>• Sibling of a child/adolescent/young adult who is/was in treatment for cancer<br>• Patient-Advocate<br>• Other: _________<br><br>**Which is the disease in question?**<br>(Multiple choice)<br>• Oncological disease<br>○ Leukemia, lymphoma or other hematological cancer<br>○ Brain tumour<br>○ Other solid tumour<br>• Endocrinological disease<br>• Hematological disease<br>• Neurological disease<br>• None<br>• Other:<br>○ Cardiological disease<br>○ Nephrological disease<br>○ Dermatological disease<br>○ Orthopaedic disease<br>○ Pneumological disease<br>○ Urological disease<br>○ Visceral disease

- Gastroenterology
- Geriatrics
- HNO
- Internal medicine
- Cardiology
- Nephrology
- Neurology
- Endocrinology
- Orthopaedics
- Paediatrics
- Palliative care
- Pneumology
- Radiology and Nuclear Medicine
- Accident surgery
- Urology
- Visceral surgery
- Dentistry, Oral and Maxillofacial Medicine

My setting:

(Multiple choice)

- Acute Treatment
- Rehabilitation
- Follow-Up Care
- Research
- Care
- Other: _______

For how long have you been working in this field?

- 0-4 years
- 5-9 years
- 10-14 years
- 15-20 years
- >20 years

This is how research and care are distributed in my working-routine:

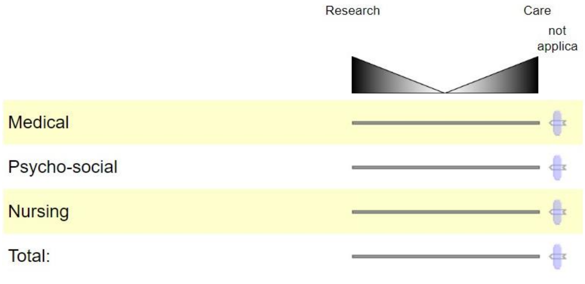

Age at time of diagnosis:

I was ______ years old.

The sick person was _____ years old. (If applicable)

How was the disease treated?

(Multiple choice)

- Surgery
- Radiotherapy
- Chemotherapy
- Immunotherapy
- Other: _______

Instructions for Patient Advocates

In case you are a **Patient advocate/representative**, without being directly affected by paediatric cancer yourself (as a patient, survivor, parent, sibling etc.), please proceed like this: **Check "Patient Advocate" and also check "Other"** and indicate that zou are not personally affected, as well as the field that you are advocating for (e.g. paediatric oncology). You can omit the question about the specific illness and the treatment. Note: If you click on "next", you will be asked if you are sure you want to continue and not leave any other replies on this side. If this is the case, check the box wiht "yes" and click "next". For all other questions please reply from your personal point of view and experience in the field.

Page 6: Current knowledge about PPIE

When I think about research and development in Pediatric Oncology / my discipline, this is what comes to my mind:

____

(Free text)

PPIE is short for „**Patient and Public Involvement and Engagement**“ in research and development, for instance the active involvement of patients or members of the public when it comes to research regarding certain diseases or the development of programs in the health sector.

<u>This is how much I know about the topic of PPIE:</u>

I am a...

- Novice (I have never heard of PPIE before)
- Beginner (I am aware of the basic idea of PPIE)
- Intermediate (I have already been in contact with PPIE)
- Proficient (I know about PPIE and can describe its aspects)
- Expert (I am well-practiced, and have versatile knowledge about PPIE)

When I think about research and development in health care (medical), this is what comes to my mind:

(Any number of answers possible)

___________________________ (free text)

This is how much I know about the topic of PPIE now:

	Not at all — Very well	n. a.
**Patient Involvement**		
**Participation**		
**Patient Engagement**		

How would you briefly describe the following three phrases in the context of PPIE in your own keywords?

Please answer the following quenstions from your <u>own perspective</u>!!

When I think about research and development in the health care sector, this is what comes to my mind:

____

(Free text)

PPIE is short for „**Patient and Public Involvement and Engagement**“ in research and development, for instance the active involvement of patients or members of the public when it comes to research regarding certain diseases or the development of programs in the health sector.

<u>This is how much I know about the topic of PPIE:</u>

I am a...

- Novice (I have never heard of PPIE before)
- Beginner (I am aware of the basic idea of PPIE)
- Intermediate (I have already been in contact with PPIE)
- Proficient (I know about PPIE and can describe its aspects)
- Expert (I am well-practiced, and have versatile knowledge about PPIE)

When you think about research and development: Where / How can patients be involved?

(Any number of answers possible)

___________________________ (free text)

This is how much I know about the topic of PPIE now:

	Not at all — Very well	n. a.
**Patient Involvement**		
**Participation**		
**Patient Engagement**		

Patient Involvement

- ____________________ (free text)
- n. a.

Participation

- ____________________ (free text)
- n. a.

Patient Engagement

- ____________________ (free text)
- n. a.

How would you briefly describe the following three phrases in the context of PPIE in your own keywords?

Patient Involvement

- ____________________ (free text)
- n. a.

Participation

- ____________________ (free text)
- n. a.

Patient Engagement

- ____________________ (free text)
- n. a.

Page 7: Possibilities for involvement

When you think about research and development: Where / How can patients be involved?

(Multiple choice)

- As study participant
- Answering questionnaires
- Reviewing patient-brochures
- Reviewing patient information and informed consent papers
- Reviewing submissions to ethics committees
- Co-Planning design and equipment of health care facilities (e.g. when a new hospital or ward is being built etc.)
- User ratings of research results (e.g. of services or programs for patients and their families, new treatment methods etc.)
- Research funding (e.g. raise funds / donations for research projects)
- Setting research priorities
- Defining research questions
- Development of patient brochures
- Development of patient information and informed consent papers
- Development of research-/project proposals
- Development of ethics proposals
- Development of treatment-protocols
- Development of study reports / data analysis
- Post-study communication
- Contribution to publications
- Dissemination of research results to patient community
- As members of steering committees
- As members of data-safety / monitoring committees
- Participation in investigator-meetings
- Not at all
- Other: ____________

Page 8: Perceived relevance of research

<table>
<tr>
<td>
<u>Please indicate an assessment according to your personal experience regarding the following statements:</u>

In my opinion, medical research is...
Not important — Important
A burdening additional effort — An integral part
Irrelevant for patient care — Relevant for patient care

In my opinion, medical psycho-social is/was:
Not important — Important
A burdening additional effort — An integral part
Irrelevant for patient care — Relevant for patient care

In my opinion, nursing research is/was:
Not important — Important
A burdening additional effort — An integral part
Irrelevant for patient care — Relevant for patient care

Please indicate how important PPIE in research and development is in your opinion:
Not important — Important
A burdening additional effort — An integral part
Irrelevant for patient care — Relevant for patient care
</td>
<td>
Please answer the following quenstions from your <u>own perspective!</u>!

<u>Please indicate how important research and development are/were during treatment:</u>

If you are a family member or a patient advocate who is not personally affected, please still answer the question from your personal point of view and experience.

In my opinion, medical research is...
Not important — Important
A burdening additional effort — An integral part
Irrelevant for patient care — Relevant for patient care

In my opinion, medical psycho-social is/was:
Not important — Important
A burdening additional effort — An integral part
Irrelevant for patient care — Relevant for patient care

In my opinion, nursing research is/was:
Not important — Important
A burdening additional effort — An integral part
Irrelevant for patient care — Relevant for patient care

Please indicate how important PPIE in research and development is in your opinion:
Not important — Important
A burdening additional effort — An integral part
Irrelevant for patient care — Relevant for patient care
</td>
</tr>
<tr>
<td colspan="2">Page 9: Subjective experience with PPIE</td>
</tr>
<tr>
<td>
I have been involved in the following research fields:
(Multiple choice)
• Medical research
</td>
<td>
Please answer the following quenstions from your <u>own perspective!</u>!

I have been involved in the following research fields:
</td>
</tr>
</table>

- Psycho-social research
- Nursing research
- None
- Other

There are different roles patients can adopt when being involved or participating in research. Which roles have the patients you have already worked with taken on?

(Multiple choice)

- **"Individual Patient / Carer":** personal experience of living with a disease"
- **"Patient Advocate"** : Experiences with big(ger) groups of patients"
- **"Patient Organization Representative"** : Perspectives of patient organizations"
- **"Patient Expert"** : Additional professional expertise, e.g. in the field of research & development, health policies etc.

To what extent are you involving your patients in the following phases of research and development:

	Not at all – Fully	n. a.
Defining the research questions:		
Study Design		
Data collection		
Data analysis		
Communication of results		
Implementation of results into daily practice		
Study participant		

Where would you like to involve patients?

(optional)

___________________________ (free text)

And why?

(optional)

___________________________ (free text)

(Multiple choice)

- Medical research
- Psycho-social research
- Nursing research
- None
- Other

There are different roles patients/care givers/ representatives can adopt when involved or participating in research. In which role would you see yourself?

(Multiple choice)

- **"Individual Patient / Carer":** personal experience of living with a disease"
- **"Patient Advocate"** : Experiences with big(ger) groups of patients"
- **"Patient Organization Representative"** : Perspectives of patient organizations"
- **"Patient Expert"** : Additional professional expertise, e.g. in the field of research & development, health policies etc.

To what extent are you involved / were you involved in the following phases of research and development:

	Not at all – Fully	n. a.
Defining the research questions:		
Study Design		
Data collection		
Data analysis		
Communication of results		
Implementation of results into daily practice		
Study participant		

Where would you like to be involved?

(optional)

___________________________ (free text)

And why?

(optional)

<table>
<tr>
<td>Which challenges or concerns come to your mind when you think about involvement of patients in research and development according to the concept of PPIE?
(Any number of answers possible)
_________________________ (free text)</td>
<td>_________________________ (free text)

Which challenges or concerns come to your mind when you think about involvement of patients in research and development according to the concept of PPIE?
(Any number of answers possible)
_________________________ (free text)</td>
</tr>
<tr>
<td colspan="2">Page 10: Knowledge about PPIE</td>
</tr>
<tr>
<td colspan="2">ALMOST DONE! This is the last page

<u>This is how much I know about the topic of PPIE now:</u>
I am a...
• Novice (I have never heard of PPIE before)
• Beginner (I am aware of the basic idea of PPIE)
• Intermediate (I have already been in contact with PPIE)
• Proficient (I know about PPIE and can describe its aspects)
• Expert (I am well-practiced, and have versatile knowledge about PPIE)

What I have always wanted to say about the topic of patient involvement:
(optional)
_________________________ (free text)</td>
</tr>
<tr>
<td colspan="2">Page 10: Contact information</td>
</tr>
<tr>
<td colspan="2"><u>You made it!</u>
Thank you so much for participating in our survey! Your answers will help in better and more purposefully implementing patient involvement in the future!
If you have any further questions about our project, do not hesitate to get in touch with us via these e-mail addresses: xxxxx</td>
</tr>
</table>
